# Effect of retinol and α-tocopherol supplementation on photoreceptor and retinal ganglion cell apoptosis in diabetic rats model

**DOI:** 10.1186/s40942-022-00392-2

**Published:** 2022-06-17

**Authors:** Andi Muhammad Ichsan, Agussalim Bukhari, Subehan Lallo, Upik Anderiani Miskad, Andi Afdal Dzuhry, Itzar Chaidir Islam, Habibah Setyawati Muhiddin

**Affiliations:** 1 Department of Ophthalmology, Faculty of Medicine, Hasanuddin University, Makassar, Indonesia; 2 Department of Clinical Nutrition, Faculty of Medicine, Hasanuddin University, Makassar, Indonesia; 3 Department of Pharmaceutical Science, Faculty of Pharmacy, Hasanuddin University, Makassar, Indonesia; 4 Department of Pathology, Faculty of Medicine, Hasanuddin University, Makassar, Indonesia

**Keywords:** Diabetic retinopathy, Retinol, α-tocopherol, Photoreceptor cell, Retinal ganglion cell, Apoptosis

## Abstract

**Background:**

Diabetic retinopathy (DR) is the most common microvascular complication of diabetes. Retinol and α-tocopherol of diabetic models prevent the damage of photoreceptor and retinal ganglion cells (RGC) caused by hyperglycemia.

**Objective:**

This study aims to examine the effect of retinol and α-tocopherol on photoreceptor and RGC densities and the expression of caspase-3 and -7 on the retinal layers of the diabetic rat model.

**Methods:**

Alloxan 150 mg/kg body weight single dose was used to develop animal models, which were separated into eight groups. These consist of one group without intervention (group 1), one positive control with only induced alloxan (group 2), and others receiving retinol (group 3 and 6), α-tocopherol (group 4 and 7), or their combination (group 5 and 8). Furthermore, histopathological examination was performed using Hematoxylin–Eosin staining to evaluate the photoreceptor and RGC densities, while immunohistochemistry staining evaluated the caspase-3 and -7 expressions.

**Results:**

In the treatment group, the highest and lowest densities were identified in diabetic rats given α-tocopherol (group 7) and retinol (group 3) respectively. The caspase-3 and -7 expression showed that the group given α-tocopherol (group 7) had the lowest value.

**Conclusion:**

In diabetic rats, retinol and α-tocopherol compounds maintained densities and prevented photoreceptor and RGC death. However, α-tocopherol was more promising than retinol or combinations in the prevention of retinal cells apoptosis.

## Background

Diabetic retinopathy (DR) is one of the typical causes of visual impairment in the productive-age class worldwide [40]. Based on the abnormalities of the retinal microvasculature, DR is a microvascular complication of diabetes. However, a recent pathophysiological model has highlighted that neurodegeneration is a crucial and early component of this complication. Neural apoptosis, response gliosis, glutamate excitotoxicity, the decline in neuroprotective components, and debilitation of the neurovascular coupling are depicted as causes of retinal neurodegeneration. One of the underlying pathomechanisms for DR found to precede visible vasculopathy is neurodegeneration [13]. Previous study showed that the neuronal unit of the retina and DR are strongly related because retinal neurons and glial cells demonstrate biochemical defects and functional abnormalities. This involves rapid neuronal death, microglial cell activation, and enhanced oxidative stress generation by photoreceptors [19].

The most often utilized diabetogenic drugs are alloxan and streptozotocin [12]. Alloxan is a highly potent diabetogenic cyclic-urea derivative that can generate reactive oxygen species (ROS) in a cyclic reaction with its reduction product, dialuric acid, in the presence of intracellular thiols, particularly glutathione. The beta cell toxicity is begins by the free radicals produced during the redox reaction. Autoxidation of dialuric acid produces superoxide radicals (O2-), hydrogen peroxide (H2O2), and hydroxyl radicals in a final iron-catalyzed reaction step (OH-). These hydroxyl radicals ultimately cause beta cells to die due to their innately limited ability for antioxidative defense and the resulting state of insulin-dependent alloxan diabetes. As a thiol reagent, alloxan inhibits glucose-induced insulin secretion selectively by oxidizing important thiol groups in the glucokinase protein, disrupting oxidative metabolism and this beta-cell signaling enzyme [22]. The dose used to cause diabetes varies between 40 and 200 mg/kgBW through the intraperitoneal or intravascular route [38].

Caspases involved in apoptosis have been subclassified by their mechanism of action and are either initiator (caspase- 8 and -9) or executioner (caspase-3, -6, and -7) [25]. Caspase-3 with -7 are similar because the cysteine proteases share an optimal peptide recognition sequence and have several endogenous protein substrates in common. In addition, they are proteolytically activated by the initiator caspase-8 and -9 during death receptor- and DNA-damage-induced apoptosis [20]. Caspase-3 and -7 expression as apoptotic markers might be used to investigate the alterations in the retina following the diabetes condition. These are two of the essential caspase effectors in apoptotic pathways, and the indicators can assess the level of tissue damage caused by the induction agent [18, 20].

The amounts of damaged DNA and nitrosylated proteins are higher in the diabetic retina due to increased oxidative stress (OS) and compromised antioxidant defense enzymes. Diabetic experimental animals and humans have a lower level of antioxidant enzymes and vitamins [27]. Antioxidants can be used to alleviate metabolic and functional abnormalities as a result of the close relationship between OS and dysmetabolism associated with the pathogenesis of DR. They can work on various levels, such as inhibiting the generation of reactive oxygen species (ROS), lowering free radicals, or enhancing enzyme capacities. The finding demonstrated that medicinal and aromatic plants' dietary or local bio factors could help manage diabetes. OS triggers other unfavorable pathways to DR development and causes a vicious circle of injury to macromolecules by magnifying additional ROS. Therefore, OS and ROS are considered to have a role in DR by increasing glucose and significant metabolic abnormalities [24, 39].

The extensive investigation of vitamins A, C, E, and carotenoids are well-known antioxidants produced from food. Antioxidants can limit the generation of reactive oxygen species (ROS), scavenge free radicals, or boost the enzyme capabilities to reduce oxidative stress-induced damage to the retina [39].

Retinol, retinal, retinoic acid, and provitamin A carotenoids are unsaturated nutritional chemical molecules that make up vitamin A [43]. Painstaking biochemical reconstitution experiments have enabled recent improvements in the molecular knowledge of the retinoid cycle in the mammalian retina. Furthermore, natural or synthetic animal models with known genetic lesions backed this claim with human studies of target genetic blinding diseases. Critical retinal enzymes and proteins as well as their substrates and ligands have been identified using structural and membrane biology in a cellular context [17]. In a reversible reaction catalyzed by the reduced nicotinamide adenine dinucleotide phosphate (NADPH) -dependent all-trans-retinol dehydrogenase, all-trans-retinal in the cytoplasm were degraded to all-trans-retinol. This product diffuses into the retinal pigment epithelium, which is esterified by lecithin retinol acyltransferase (LRAT) [30]. Meanwhile, in vitro and in vivo studies showed a protective impact of α-tocopherol, a vitamin E derivative, on eye tissues. For up to 24 h of exposure, a biomolecular compound of α-tocopherol can protect the retina against light damage [34]. Therefore, this study aimed to investigate the protective effect of retinol and α-tocopherol on the photoreceptor and retinal ganglion cells apoptosis in a diabetic rats model.

## Methods

### Design

This study was conducted with a post-test group of forty animal subjects at the Animal and Pathology Laboratories of Hasanuddin University, Indonesia. This study received approval from The Ethics Committee of Medical Research, Faculty of Medicine, Hasanuddin University, with number: 725/UN4.6.4.5.31/PP36/2021.

Alloxan monohydrate (SIGMA USA, Cat. No. A7413) 150 mg/kgBW single dose intraperitoneally was used to induce the diabetic model. Supplementation was performed with retinol (SIGMA USA, Product No. R7632, CAS Number: 68-26-8) and α-tocopherol (SIGMA USA, Cat. No.258024) compounds. Furthermore, eight groups of animals were created, where group 1 was the negative control (wild type), group 2 was the positive control (alloxan induction without treatment), group 3 described diabetic rats on retinol for 1 week (after alloxan induction), group 4 represented the diabetic rats on α-tocopherol for 1 week (after alloxan induction), group 5 was the sample given a combination of retinol and α-tocopherol for 1 week (after alloxan induction), group 6 represented diabetic rats on retinol for 14 days (1 week each, before and after alloxan induction), group 7 described the samples given α-tocopherol for 14 days (1 week each, before and after alloxan induction), and group 8 was the combination of retinol and α-tocopherol for 14 days (1 week each, before and after alloxan induction).

### Established animal experiment

Male Wistar rats (*Rattus norvegicus*) of 8–12 weeks old, weighing 160–200 g, were used for this study. All animals were given standard feed and access to ad libitum drinking water in a room with a 12-h light–dark cycle. Each experimental animal in groups 2–8 received 150 mg/kg body weight an intraperitoneal injection of Alloxan monohydrate. Induction was considered successful where blood glucose levels were > 200 mg/dl. Furthermore, blood sugar measurements were performed three times before alloxan injection, 3 days later, and a day before sacrifice. All samples in group 1 had a blood glucose level < 200 mg/dl, while those in groups 2 to 8 had a blood glucose level > 200 mg/d after being induced. Retinol compounds up to 900 mcg/day were administered to groups 3 and 6 [26]. α-tocopherol compounds up to 15 mg/day were also provided to groups 4 and 7 [33], while 5 and 8 received both.

### Sample collection and processing

The rats were sacrificed before enucleation by placing in a closed container filled with cotton and ether for approximately 10 min until there was no motoric reaction, neurological reflexes, or heartbeat. Subsequently, the eye tissue was removed using the enucleation approach, which involved pressing the eyeball on the base of the optic nerve, cutting the optic nerve, and lifting the eyeball. Finally, all eyes were fixed with 10% formalin and transported to the pathology laboratory.

Retinal tissue was cut using a microtome with a thickness of 5 µm and stained with hematoxylin and eosin (HE) to calculate the density of ganglion and photoreceptor cells. Caspase-3 (Cat No. C9598, Sigma USA) and -7 (Cat No. C1104, Sigma USA) expression in the retinal layer was examined using immunohistochemistry (IHC). Quantitative approaches were used to interpret cell density using an Olympus CX23 binocular microscope with 40-fold objective magnification, and the results were expressed as a mean with standard deviation. Immunohistochemistry staining was conducted using a primary and secondary antibody (Cat. No. UCS015-IFU, ScyTek USA) to identify caspase-3 and -7. The intensity of expression in photoreceptor cells was categorized qualitatively using the Immunoreactive Scoring System (IRS) modification method. The three categories are negative (caspase expression shows < 5%), low (5–20% expression), and high (> 20% expression). Meanwhile, the intensity of caspase expression in retinal ganglion cells was calculated quantitatively by counting the number of cells and apoptotic bodies.

### Data analysis

Statistical analysis used an Independent T-test and Kruskal Wallis for the quantitative and qualitative data (sig. p < 0.05).

## Results

According to Table 1, the blood sugar level of the negative control was 82 ± 2 mg/dl compared to the diabetic groups (276 ± 15 to 426 ± 45 mg/dl). This showed that the experimental animal could be used as a model for type 1 diabetes rats because they have hyperglycemic conditions.

**Table 1:** Descriptive data

Group	Treatment	Photoreceptor cells density (mean ± SD)	p-value	Ganglion cells density (mean ± SD)	p-value	Caspase-3 expression on Photoreceptor cells (n)	p-value	Caspase-3 expression on Ganglion cells (mean ± SD)	p-value	Caspase-7 expression on Photoreceptor cells (n)	p-value	Caspase-7 expression on Ganglion cells (mean ± SD)	p-value	Blood glucose level (mean ± SD)
1	Negative control (Wild type)	843 ± 32	**0.002**^**a**^	26 ± 1	**0.010**^**a**^	High: 0 Low: 2 Neg: 3	**0.016**^**b**^	16.80 ± 3.701	**0.010**^**a**^	High: 0 Low: 3 Neg: 2	**0.069**^**b**^	18.60 ± 1.94	**0.010**^**a**^	82 ± 2
2	Alloxan induction without treatment	565 ± 95		19 ± 3		High: 4 Low: 1 Neg: 0		76.20 ± 14.97		High: 2 Low: 3 Neg: 0		77.20 ± 10.82		276 ± 15
3	Retinol for 7 days	536 ± 138		18 ± 5		High: 2 Low: 3 Neg: 0		33.80 ± 5.16		High: 2 Low: 3 Neg: 0		44.80 ± 25.78		349 ± 63
4	α-tocopherol for 7 days	701 ± 88		25 ± 5		High: 1 Low: 4 Neg: 0		32.80 ± 2.28		High: 2 Low: 3 Neg: 0		27.20 ± 3.27		426 ± 45
5	Combination of retinol and α-tocopherol for 7 days	614 ± 156		20 ± 4		High: 3 Low: 2 Neg: 0		43.00 ± 10.70		High: 3 Low: 2 Neg: 0		40.60 ± 8.29		400 ± 83
6	Retinol for 14 days (pre and post induction)	599 ± 93		20 ± 5		High: 3 Low: 2 Neg: 0		37.00 ± 3.53		High:3 Low: 2 Neg: 0		44.80 ± 4.60		387 ± 81
7	α -tocopherol for 14 days (pre and post alloxan induction)	752 ± 190		27 ± 2		High: 1 Low: 4 Neg: 0		38.60 ± 7.79		High: 2 Low: 3 Neg: 0		32.40 ± 4.50		404 ± 66
8	Combination of retinol and α-tocopherol for 14 days (pre and post alloxan induction)	657 ± 78		25 ± 5		High: 3 Low: 2 Neg: 0		48.20 ± 6.64		High: 3 Low: 2 Neg: 0		41.40 ± 3.84		424 ± 52

^a^One-way ANOVA test (sig < 0.05)

^b^Kruskal–Wallis test (sig < 0.05)

**Fig. 1.**
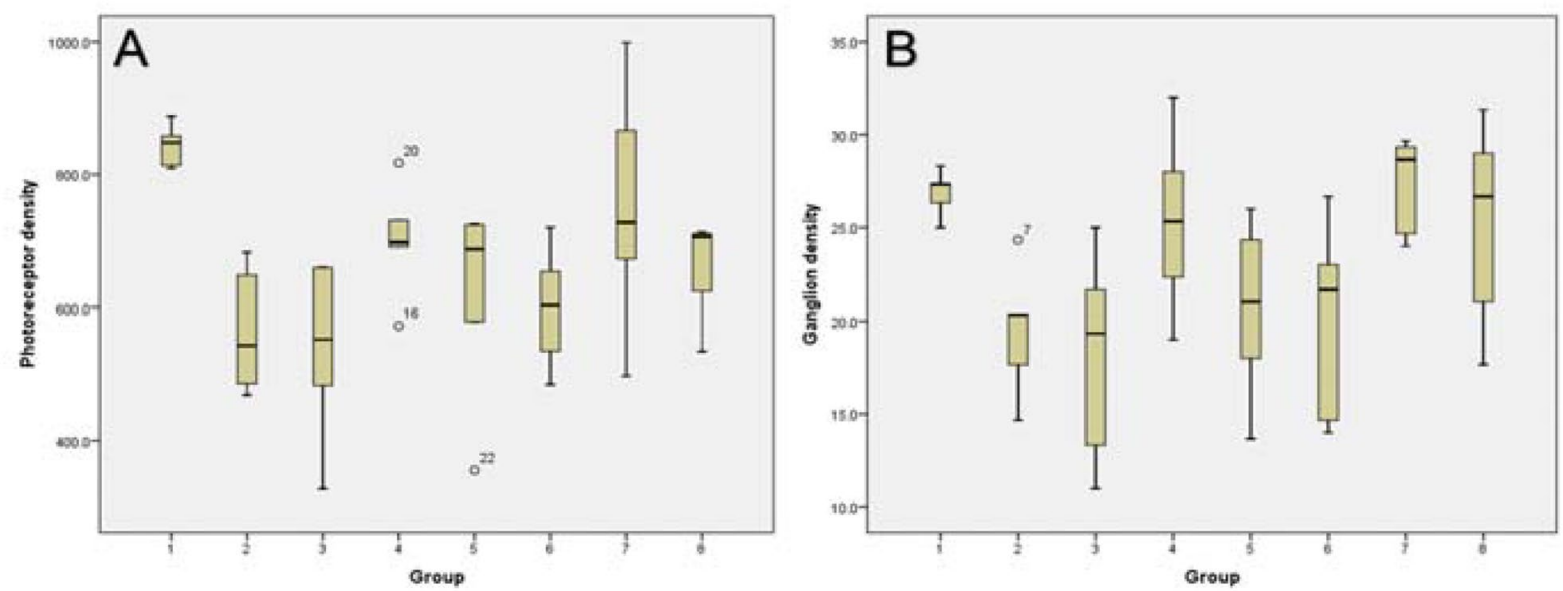
**A** Photoreceptor cells density; **B** Ganglion cells density

The photoreceptor cell density showed the highest and lowest value in groups 1 and 2, respectively. In the treatment group, the most effective value for approaching the normal group of mice was in group 7, which received α-tocopherol supplementation for 14 days (pre and post-alloxan induction). The statistical test results showed a significant difference in the photoreceptor cell density among groups (p = 0.002), as shown in Fig. 1a. This result is in line with the measurement of retinal ganglion cell density, where the highest and lowest value was also obtained in groups 1 and 2, respectively. For the treatment group, the most effective supplementation was shown in group 7 (Fig. 1b). In addition, the statistical test result showed that there was a significant difference in the RGC density among groups (p = 0.010).

**Fig. 2.**
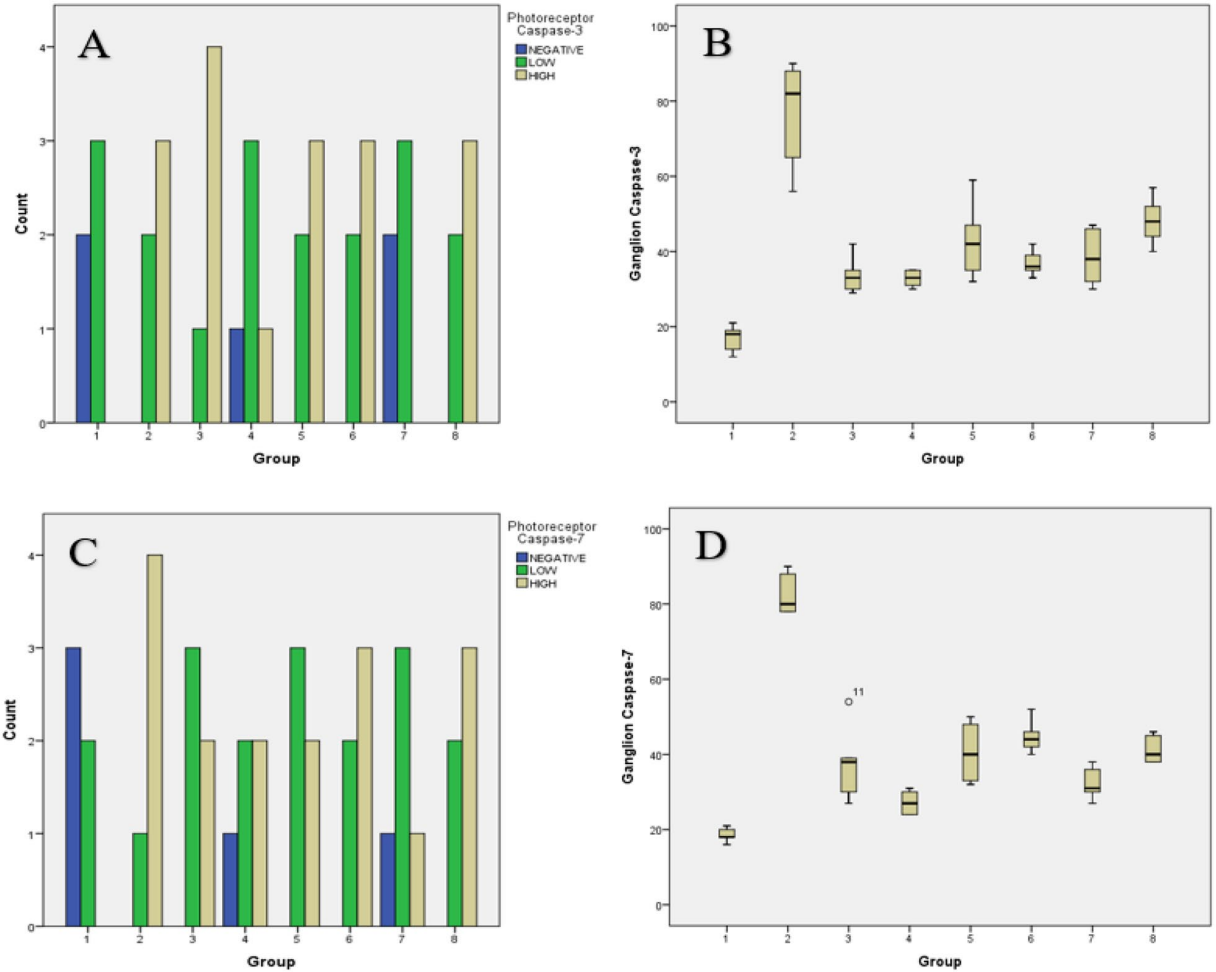
**A** Caspase-3-expression on photoreceptor cells. **B** Caspase-3-expression on ganglion cells; **C** Caspase-7-expression on photoreceptor cells. **D** Caspase-7-expression on ganglion cells

In calculating the expression of caspase-3, apoptosis in photoreceptor cells showed the lowest and highest expression in groups 1 and 2, respectively. Values close to the normal were shown by groups 4 and 7 (α-tocopherol supplementation groups), as presented in Fig. 2a. Statistical analysis showed a significant difference in the difference caspase-3 expression in photoreceptor cell among groups (p = 0.016).

The above results are in line with the those of expression in retinal ganglion cells, where the lowest and highest value was found in groups 1 and 2, respectively. The observation results in the treatment group were found to be the most effective in the group given α-tocopherol supplementation (groups 4 and 7), as shown in Fig. 2b. Statistically, these results showed a significant caspase-3 expression in RGC among groups (p = 0.010).

The expression value of caspase-7, apoptosis in photoreceptor cells showed the lowest in group 1, while the highest was found in 5, 6, and 8. Observations in the treatment group indicated that groups 4 and 7 (supplementation of α-tocopherol) showed the lowest expression. Based on these results, it was obtained that this result did not significantly affect caspase-7 expression in photoreceptor cell among groups (p = 0.069). The value of the expression in retinal ganglion cells showed the lowest and highest value in groups 1 and 2, respectively. In the treatment group, the expression values close to the standard were samples in 4 and 7, respectively. Figures 2c and d indicated a significant difference between caspase-7 expression in RGC among groups (p = 0.010).

## Discussion

The retina is a weak and thin layer of tissue that originates from the neuroectoderm, comprising of nine layers of sensory neurons in the visual pathway [9]. Photoreceptors are visual system sensors that transform photon capture into a nerve signal through a process known as phototransduction. Photoreceptor terminals interrelate with surrounding photoreceptors and interneurons of horizontal and bipolar cells. They are required for transmitting visual information and early processing in the retina [7].

Photoreceptors in the healthy retina are among the most active oxygen consumers in the body, and the choroidal circulation supplies the majority of the oxygen to photoreceptors. As a result, oxygen tension drops quickly from the Bruch’s membrane to the retina’s outer nuclear layer, where it reaches the lowest values. This reduces oxygen reserve in photoreceptors, and even a minor disruption of oxygen flow in diabetes can result in severe hypoxia. The creation of acellular capillaries, capillary blockage, and capillary dropout can contribute to retinal hypoxia, hence, the vascular pathology of DR [3].

On the other layer, retinal ganglion cells process and convey information from the retina to visual centers in the brain. These output neurons comprise subpopulations with distinct structures and functions [37]. As a result, there is a remarkable diversity of RGCs. The various subtypes have unique morphological features and pathways linking the inner retina to the relevant brain areas [16]. Retinal ganglion cells carry visual signals from the eye to the brain but do not make chemical synapses with other neurons. However, they form gap junctions with other RGCs and amacrine cells, allowing RGC signals to feedback into the inner retina [41].

A pathogenic disease, such as diabetic retinopathy causes a decrease in the electrical activity of neurotransmitters from photoreceptors and RGC cells to the nerve fiber layer [2]. DR is a duration-dependent disease infrequently discovered during the early years of diabetes. However, it substantially develops with time, nearly 90% of patients showing retinopathy after 20–25 years of diabetes [19]. After cellular membranes are damaged, and intracellular components are released, oxygen-derived free radicals mediate tissue injury [29].

Antioxidants have the potential of preventing retinopathy development in diabetic rats and the implicated retinal metabolic abnormalities [39]. Therefore, to protect the retina and choroid, optimal combinations of vitamins B1, B2, B6, L-methylfolate, methylcobalamin (B12), C, D, natural α-tocopherol complex, lutein, zeaxanthin, α-lipoic acid, and n-acetylcysteine are necessary [33].

This study showed a substantial difference in cell density between diabetic and non-diabetic rats after alloxan induction as well as supplementation with retinol and α-tocopherol substances. Retinol supplementation appeared to affect maintaining the retinal cell densities positively. However, it was not better than the α-tocopherol and combination supplementation groups. The higher density values proved this in groups 3 and 6 compared with 2. The study by Zhong et al. [43] reported that retinoids might create cation radicals due to interactions with different radicals or photoexcitation with light. Furthermore, there is an indication that semi-oxidized retinoids can oxidize certain amino acids and proteins and that α-tocopherol can scavenge retinol and retinoic acid cation radicals [43].

In the retinoid cycle, retinol is an excellent substrate for LRAT and quickly converted into fatty acid esters. Their propensity to form oil droplets excludes fatty acid esters from circulation [17]. The mechanism of vitamin A transport is mediated by the plasma retinol-binding protein (RBP), a specific and sole carrier in the blood. The specific membrane receptor stimulated by retinoic acid 6 (STRA6) mediates cellular vitamin A uptake. [43] Structural and membrane biology have been used to detect critical retinal enzymes and proteins as well as their substrates and ligands, placing them in a cellular context. The most presently accepted modulators of the retinoid cycle have demonstrated promising results in animal models of retinal degeneration [17].

The α-tocopherol supplementation group was closest to the normal values for photoreceptor and retinal ganglion cell densities. A similar result was found in Ritch [34], which stated that the α-tocopherol had been suggested to protect against retinal phototoxicity and central nervous system ischemia [34]. Once the fat is oxidized and free radical reactions propagate, α-tocopherol is a powerful chain-breaking antioxidant that counteracts reactive oxygen species molecules creation. By inhibiting the peroxidation of membrane lipids and scavenging lipid peroxyl radicals, it protects essential cellular structures from damage produced by oxygen free radicals and reactive products of lipid peroxidation [14]. This also protects the polyunsaturated fatty acids found in membrane phospholipids and plasma lipoproteins because of its peroxyl radical scavenging activity [35].

Vitamin E refers to eight naturally occurring compounds (α-, β-, γ-, δ-tocopherol, and α-, β-, γ-, δ-tocotrienol). α-tocopherol is the most common form retained in human plasma out of the eight forms [8]. Vitamin E is crucial for erythrocytes’ stability as well as central and peripheral nerves conductivity. Therefore, several countries have established dietary vitamin E recommendations [32].

**Fig. 3.**
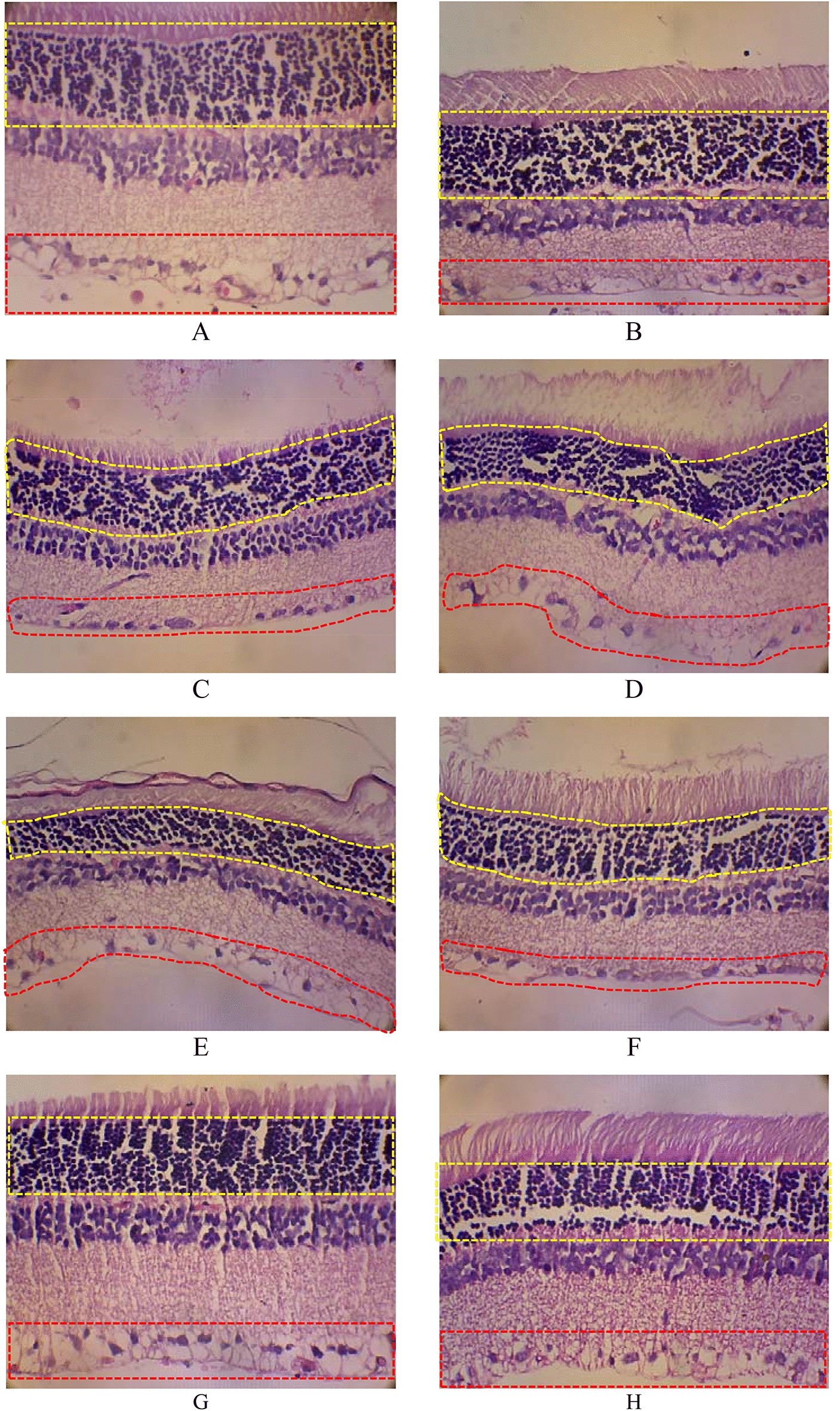
HE staining for cell density**.** **A** = group 1, **B** = group 2, **C** = group 3, **D** = group 4, **E** = group 5, **F** = group 6, **G** = group 7, **H** = group 8; Yellow line: photoreceptor cell’s nucleus; Red line: retinal ganglion cells.

The loss of photoreceptors in the diabetic retina is still debatable, and various optical coherence tomography (OCT) studies in diabetic patients show that the thickness of the inner retina, including the nerve fiber, retinal ganglion cell, and inner plexiform layers, decreases with the duration of diabetes [3]. In a diabetic animal model study, the outer nuclear layer thickness is frequently reduced, specifically in models of type 1 disease with early-onset. Furthermore, various studies supported the notion that photoreceptor loss increases with disease duration [15]. A similar condition was found, where cell densities significantly decreased in group 2 compared to others in photoreceptor and retinal ganglion cells (Fig. 3).

A high dose of α-tocopherol following positive results in a diabetic rat model to prevent diabetes-related vascular damage was examined in the clinic for administration. This was performed to restore retinal blood flow in diabetic type I patients, which was discovered to control levels. Furthermore, the α-tocopherol is useful in DR by the nonenzymatic free radical scavenging action outside the cell. Antioxidant therapy with α-tocopherol has been shown in humans to improve retinal vascular hemodynamics [39].

In this study, the administration of the combination of retinol and α-tocopherol did not show any more effective results to maintain the retinal cell densities than the single supplementation of α-tocopherol. A previous study reported that supplementation with substantial doses of retinol was demonstrated to reduce the bioavailability in growing pigs and calves [11]. Due to its abundance in human and animal tissues, α-tocopherol is a significant contributor to dietary lipid peroxidation in vivo. As a result, there have been various investigations about its effects on lipid peroxidation and combinations with other antioxidants [42].

A resonance-stabilized phenoxyl radical is created during the α-tocopherol donation of electrons. This has a reduced reaction compared to lipid-derived peroxyl radicals and does not quickly reproduce the radical chain in lipid peroxidation. Subsequently, certain biological reductants, such as ascorbate (vitamin C), ubiquinol, or dihydrolipoic acid, convert the tocopherol radical back to tocopherol. Retinoids’ interaction with hydroxyl radicals, peroxyl radicals, such as trichloromethyl peroxyl radical, or the photoionization of retinoids by exposure to ultraviolet light is responsible for the cation radicals production [6].

**Fig. 4.**
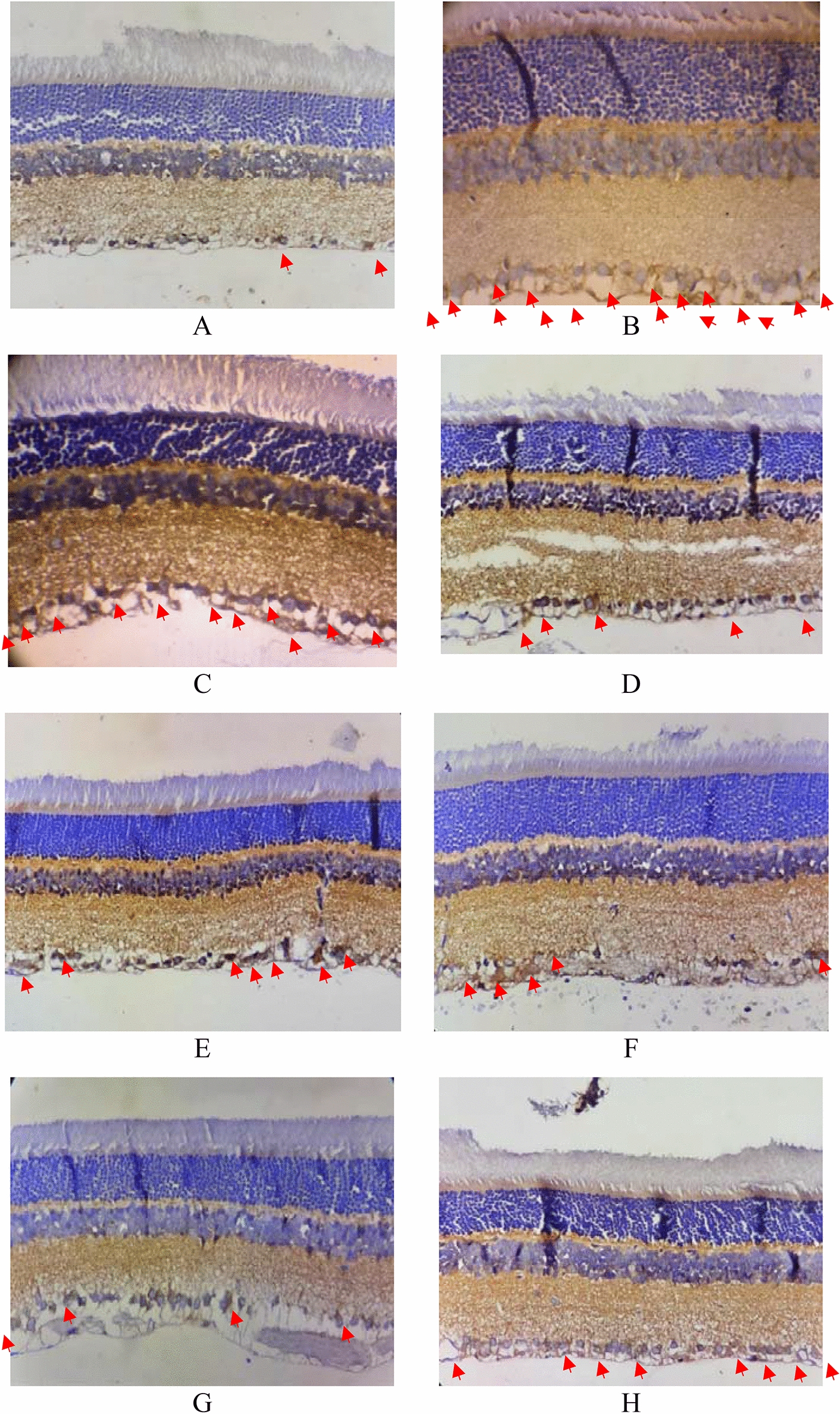
Immunohistochemistry staining for Caspase-3 expression. **A** = group 1, **B** = group 2, **C** = group 3, **D** = group 4, **E** = group 5, **F** = group 6, **G** = group 7, **H** = group 8. The red arrow shows caspase 3

**Fig. 5.**
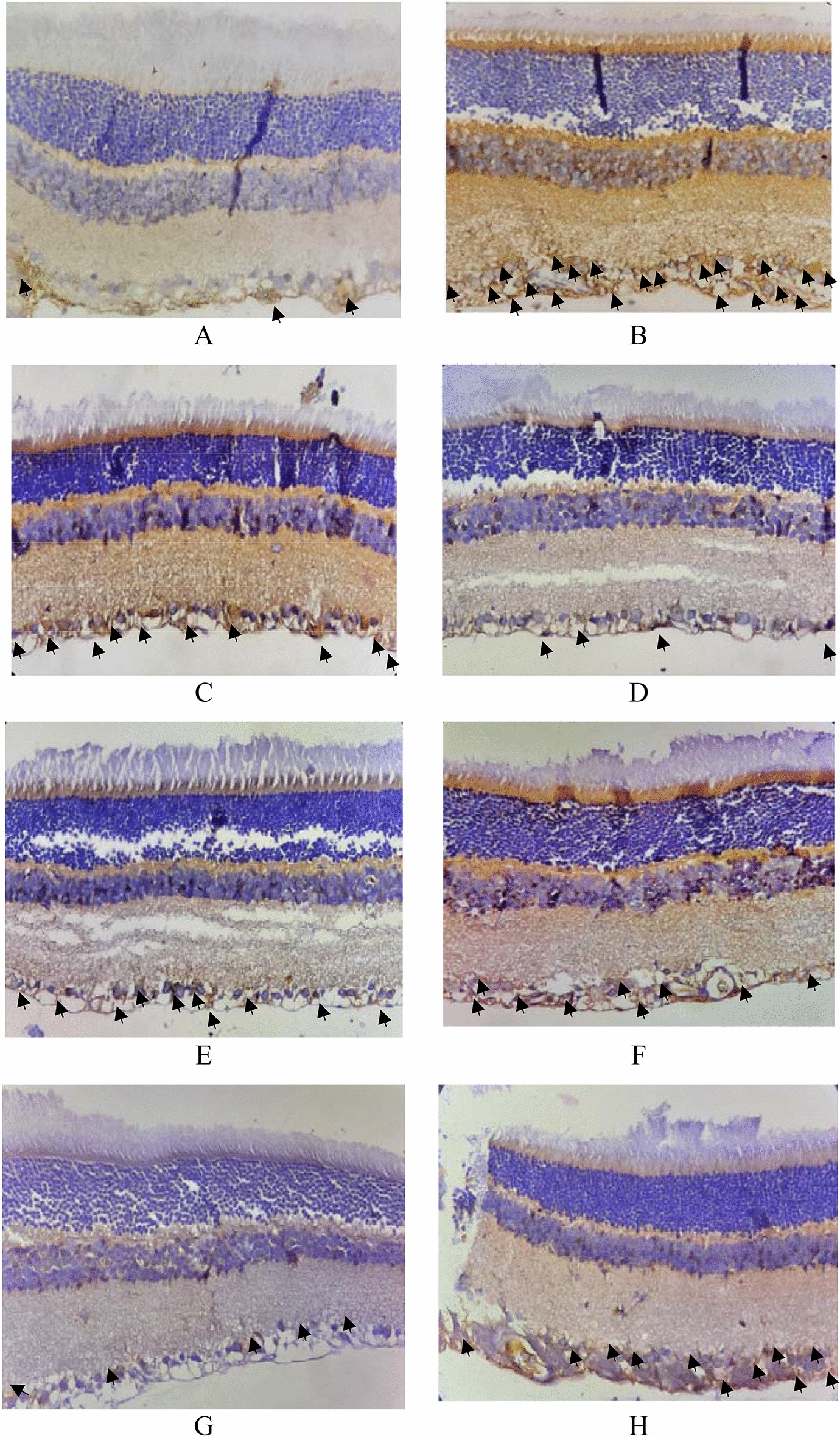
Immunohistochemistry staining for Caspase-7 expression. **A** = group 1, **B** = group 2, **C** = group 3, **D** = group 4, **E** = group 5, **F** = group 6, **G** = group 7, **H** = group 8. The black arrow shows caspase 7

The apoptosis can be characterized by the expression of biochemical markers called caspases [21, 28]. The expression of caspase-3 and -7 (Figs. 4, 5) was also conducted in this study. Apoptosis, which called programmed cell death, is a morphologically unique process that includes cell shrinkage, cytoplasm condensation, plasma membrane blebbing, and fragmentation of chromatin and DNA into oligonucleosomes [31].

The caspases are a family of genes essential for maintaining homeostasis through regulating cell death and inflammation. This biomarker produces active signaling molecules that aid in apoptosis and are divided into two types based on their modes of action, including initiator (− 8 and − 9) and executioner caspases (− 3, − 6, and − 7) [25].

Caspase-3, a key effector caspase in apoptotic pathways, is 32-kDa proenzyme that is not active. This is broken at the aspartate residue to form the p12 and p17 subunits necessary for producing the active caspase-3 enzyme. Furthermore, this is in charge of morphological and biochemical alterations during apoptosis and can be used in computing the apoptotic index [10]. Caspase-7 is also an executioner caspase that plays a critical role in optic nerve injury and retinal ganglion cell death. The inhibition might be a novel therapeutic strategy for some neurodegenerative diseases of the retina [4].

This study showed that the percentage of cell staining at each intensity level was used to grade the interpretation of caspase-3 and -7 expressions in photoreceptor cells. The degree of positivity using Immunoreactive Scoring System (IRS) modification classified Huang, et al. [10] method into negative ≤ 5%, low = 5–20%, and high ≥ 20% [10]. Moreover, quantitative measurement was obtained for the expression of the caspase in the retinal ganglion cells.

The statistical analysis found a significant difference in caspase-3 and -7 expressions among groups, and α-tocopherol groups showed a better effect on retinal cell apoptosis prevention than others. Antioxidants prevented the progression of retinopathy in diabetic rats’ retinas, which showed elevated oxidative stress. According to previous studies, apoptosis of retinal neuronal cells is increased in experimental diabetes in rats and humans. The apoptosis-induced cell death leads to ongoing neurodegeneration, where neurons are destroyed before another histopathology occurs [1]. The significant results of the α-tocopherol groups could be due to its biochemistry compound (2,7,8-trimethyl-2- (2'-carboxyethyl)-6-hydroxychroman (Ɣ-CEHC)) that suppresses cyclo-oxygenase activity with an anti-inflammatory effect [8].

This study did not show a significant effect in combination of retinol and α-tocopherol supplementation to prevent retinal cell apoptosis. It could occur because the apparent synergism between α-tocopherol and other antioxidants is based on recycling. Furthermore, α-tocopherol decreases and recycles other semi-oxidized forms such as cation radicals of vitamin A [5, 23].

The findings are comparable to the study conducted by [36] on the effects of α-tocopherol consumption on apoptosis. It was stated that α-tocopherol (10, 20, 50, or 100 μM in 0.25 M MetOH) was the only agent capable of inducing a slight statistically significant reduction in intracellular caspase-3 activity (p < 0.05). The combinations of α-tocopherol and carotenoid cleavage products (13 μg/ml) showed a high up-regulation of intracellular caspase-3 activity, and the treatment had more significant effect than carotenoid derivatives [36]. The different results were shown because they used a combination of α-tocopherol and carotenoid cleavage products. In contrast, a combination of α-tocopherol and retinol, which is pure forms of vitamin A was used. The administration of vitamins C and E reduced superoxide generation in the retina, and diabetic mice given this combination experienced a partial reduction in retinal neovascularization. The benefits of retinal cell survival become increasingly well-known once antioxidants such as ascorbic acid, acetate, α-tocopherol, Trolox cysteine, β-carotene, and selenium are consumed. The same components can also minimize lipid peroxides and prevent superoxide dismutation with catalase reduction. Therefore, it is suggested to increase the application or consumption of a broader range of antioxidants as an effective strategy to prevent retinopathy [39].

## Conclusions

Retinol and α-tocopherol compounds have a protective effect of maintaining the retinal cells’ densities and preventing the cells from apoptotic process. Moreover, the α-tocopherol compound showed better results compared to retinol compound or a combination of both. Future studies including in humans are needed to demonstrate the better understanding of α-tocopherol supplementation in preventing diabetic retinopathy progression.

## Data Availability

The data supporting these findings are available from the corresponding author upon reasonable request.

## Abbreviations

DR: Diabetic retinopathy
OS: Oxidative stress
ROS: Reactive oxygen species
LRAT: Retinol acyltransferase
IRS: Immunoreactive Scoring System
RDA: Recommended Daily Allowance
PKC: Protein kinase C
NADPH: Nicotinamide adenine dinucleotide phosphate (NADPH) oxidase
VEGF: Vascular endothelial growth factor.
IHC: Immunohistochemistry
RBP: Retinol-binding protein
STRA6: Stimulated by retinoic acid 6 (STRA6)
OCT: Optical coherence tomography
